# Laser-wakefield accelerators for high-resolution X-ray imaging of complex microstructures

**DOI:** 10.1038/s41598-019-39845-4

**Published:** 2019-03-01

**Authors:** A. E. Hussein, N. Senabulya, Y. Ma, M. J. V. Streeter, B. Kettle, S. J. D. Dann, F. Albert, N. Bourgeois, S. Cipiccia, J. M. Cole, O. Finlay, E. Gerstmayr, I. Gallardo González, A. Higginbotham, D. A. Jaroszynski, K. Falk, K. Krushelnick, N. Lemos, N. C. Lopes, C. Lumsdon, O. Lundh, S. P. D. Mangles, Z. Najmudin, P. P. Rajeev, C. M. Schlepütz, M. Shahzad, M. Smid, R. Spesyvtsev, D. R. Symes, G. Vieux, L. Willingale, J. C. Wood, A. J. Shahani, A. G. R. Thomas

**Affiliations:** 1 Center for Ultrafast Optical Science, University of Michigan, Ann Arbor, MI 48109-2099 USA; 2 Department of Materials Science and Engineering, University of Michigan, Ann Arbor, MI 48109-2099 USA; 3 Physics Department, Lancaster University, Lancaster, LA1 4YB UK; 4 The Cockcroft Institute, Keckwick Lane, Daresbury, WA4 4AD UK; 5 The John Adams Institute for Accelerator Science, Imperial College London, London, SW7 2AZ UK; 6 Lawrence Livermore National Laboratory, NIF and Photon Sciences, Livermore, CA 94550 USA; 7 Central Laser Facility, STFC Rutherford Appleton Laboratory, Didcot, OX11 0QX UK; 8 Diamond Light Source, Harwell Science and Innovation Campus, Fermi Avenue, Didcot, OX11 0DE UK; 9 Department of Physics, Lund University, P.O. Box 118, S-22100 Lund, Sweden; 10 York Plasma Institute, Department of Physics, University of York, York, YO10 5DD UK; 11 SUPA, Department of Physics, University of Strathclyde, Glasgow, G4 0NG UK; 12 Helmholtz-Zentrum Dresden-Rossendorf, Bautzner Landstraße 400, 01328 Dresden, Germany; 13 Technische Universität Dresden, 01062 Dresden, Germany; 14 Institute of Physics of the ASCR, 182 21 Prague, Czech Republic; 15 GoLP/Instituto de Plasmas e Fusão Nuclear, Instituto Superior Técnico, U.L., Lisboa, 1049-001 Portugal; 16 Swiss Light Source, Paul Scherrer Institute, CH-5232 Villigen, Switzerland; 17 ELI Beamlines, Institute of Physics of the ASCR, 182 21 Prague, Czech Republic

**Keywords:** Plasma-based accelerators, Imaging techniques, Imaging techniques

## Abstract

Laser-wakefield accelerators (LWFAs) are high acceleration-gradient plasma-based particle accelerators capable of producing ultra-relativistic electron beams. Within the strong focusing fields of the wakefield, accelerated electrons undergo betatron oscillations, emitting a bright pulse of X-rays with a micrometer-scale source size that may be used for imaging applications. Non-destructive X-ray phase contrast imaging and tomography of heterogeneous materials can provide insight into their processing, structure, and performance. To demonstrate the imaging capability of X-rays from an LWFA we have examined an irregular eutectic in the aluminum-silicon (Al-Si) system. The lamellar spacing of the Al-Si eutectic microstructure is on the order of a few micrometers, thus requiring high spatial resolution. We present comparisons between the sharpness and spatial resolution in phase contrast images of this eutectic alloy obtained *via* X-ray phase contrast imaging at the Swiss Light Source (SLS) synchrotron and X-ray projection microscopy *via* an LWFA source. An upper bound on the resolving power of 2.7 ± 0.3 *μ*m of the LWFA source in this experiment was measured. These results indicate that betatron X-rays from laser wakefield acceleration can provide an alternative to conventional synchrotron sources for high resolution imaging of eutectics and, more broadly, complex microstructures.

## Introduction

Laser-wakefield acceleration (LWFA) is a method for producing high-energy electron beams using the accelerating field structure produced in the wake of a high-power, ultrashort pulsed laser propagating through low density plasma. During wakefield acceleration, an electron bunch “surfs” on the electric wave generated by the light pressure of an intense laser pulse^1^. This wave induces a strong longitudinal electric field that remains in phase with the relativistic driver, enabling relativistic electrons to gain significant energy from the accelerating field over long distances. Due to the lack of a breakdown limit in a plasma accelerator, accelerating gradients 1000 times stronger than those produced in conventional sources can be produced^1,2^ and the generation of high energy electron beams has been demonstrated experimentally^3–9^. Additionally, in the highly nonlinear regime, electrons undergo betatron oscillations in the strong focusing fields of the wakefield, emitting a bright source of X-rays with a source size as small as one micrometer^10–12^. Betatron X-ray beams produced *via* LWFA have been shown to produce stable, bright X-ray beams capable of high resolution tomographic imaging^11,13–17^. The resultant beams have a low divergence (on the order of a few milliradians^18^) and ultrashort duration (less than 100 fs^19^), making them useful for a large range of applications across engineering, medicine, homeland security and science^11,12,14–17^. Moreover, the demonstration of micrometer scale, keV betatron X-ray beams using a single laser shot highlights the potential of these sources for imaging of complex objects in real time using high repetition rate laser systems^13^.

One exciting application for these novel X-ray sources is as a diagnostic tool for additive manufacturing processes. Laser-aided solidification is an avenue of interest in manufacturing science that requires *in situ* measurements with high spatial and temporal resolution^20,21^. Such is the case for the solidification of eutectics, in which two (or more) solid phases grow simultaneously from a parent liquid phase^22–25^. Once solidified, eutectics act as *in situ* composite materials, providing outstanding mechanical and electrical properties that are not afforded by their constituent phases alone. It is for this reason that lightweight Al-Si alloys comprise over 90% of the total Al parts produced by the United States^26^. *Irregular* eutectics such as Al-Si are composed of one faceted phase (Si) and another non-faceted (Al) phase. Due to the stiffness of the faceted phase, irregular eutectics feature a non-periodic arrangement of lamellae (fine rods or sheets of adjacent material). The interfacial dynamics underlying irregular eutectic solidification (under relatively low cooling rates) has only recently been elucidated through synchrotron-based X-ray microtomography (denoted XRT), using conventional accelerators^27^. In general, the lamellar spacing (between Al and Si phases) can be as fine as 1 *μ*m, thus requiring experimental probes that are capable of delivering high resolution information.

As noted above, synchrotron-based XRT in the micrometer range have been achieved using modern third generation light sources, such as the the beamline for TOmographic Microscopy and Coherent rAdiology experimenTs (TOMCAT) of the Swiss Light Source (SLS) at the Paul Scherrer Institut in Switzerland^28^. The TOMCAT beamline has been employed to produce high-resolution, multimodal X-ray tomographic images using monochromatic sources with energies between 8 and 45 keV, a source size of 127 *μ*m (V) × 38 *μ*m (H) (Full-Width-Half-Maximum) and a flux of (0.5 − 2) × 10^12^ photons/sec/mm^2^ ^29^. However, while conventional synchrotron light sources yield high average brightness, they are prohibitively large and expensive, limiting access to these facilities. The 1000 × stronger accelerating gradients in a LWFA enable miniaturization of the accelerator to a standard laboratory scale, potentially increasing the accessibility of advanced photon sources. And although compact synchotron sources have recently been developed^30^, laser-driven sources also have the unique capability to be co-timed to other laser-initiated events. In this way, LWFA sources can be used for so called *pump*-*probe* experiments of laser-irradiated targets^16,19^. Additionally, while the source size of newest generation conventional beamlines has been reduced to the order of 10–20 *μ*m, the resolution limit for X-ray imaging in a parallel beam geometry on these systems is dependent on the pixel size of the detector and the brightness of the source. Conversely, for a LWFA X-ray source, where the source size has been measured to be on the order of a few micrometers^11,13,15,17^, high resolution measurements are obtained using a high geometric magnification, and the resolution requirements of the detector are relaxed (see *Methods*).

In this report, we investigated the potential of laser-based X-ray sources for the imaging of solid density targets. We present a comparison between the image sharpness and resolution of raw projection images of Al-Si alloys obtained *via* conventional synchrotron X-ray phase contrast imaging at the Swiss Light Source (SLS) and X-ray projection microscopy *via* a LWFA. The former experiment was conducted *ex situ* at the TOMCAT beamline of SLS (Paul Scherrer Institut, Switzerland)^28,29^ in 2012. In these measurements, the sample was located 20 m from the source and the sample-to-detector distance was set to 11 cm for a monochromatic X-ray energy of 28 keV produced by a broad-band (Δ*E*/*E* ≈ 2%) W/Si multilayer monochromator, resulting in virtually no geometric magnification in the X-ray regime, i.e. a value of approximately 1. The X-ray radiographic image, which is produced by the absorption and refraction of the X-ray beam within the sample, was converted to visible light using a 100 *μ*m thick LuAG:Ce scintillator. The corresponding visible light image was then optically magnified by a 10x microscope objective onto the imaging chip of a pco.2000 CCD camera with 7.5 *μ*m pixel size, yielding an effective pixel size of 0.75 *μ*m. Individual images were acquired with a 500 ms exposure time.

**Figure 1:**
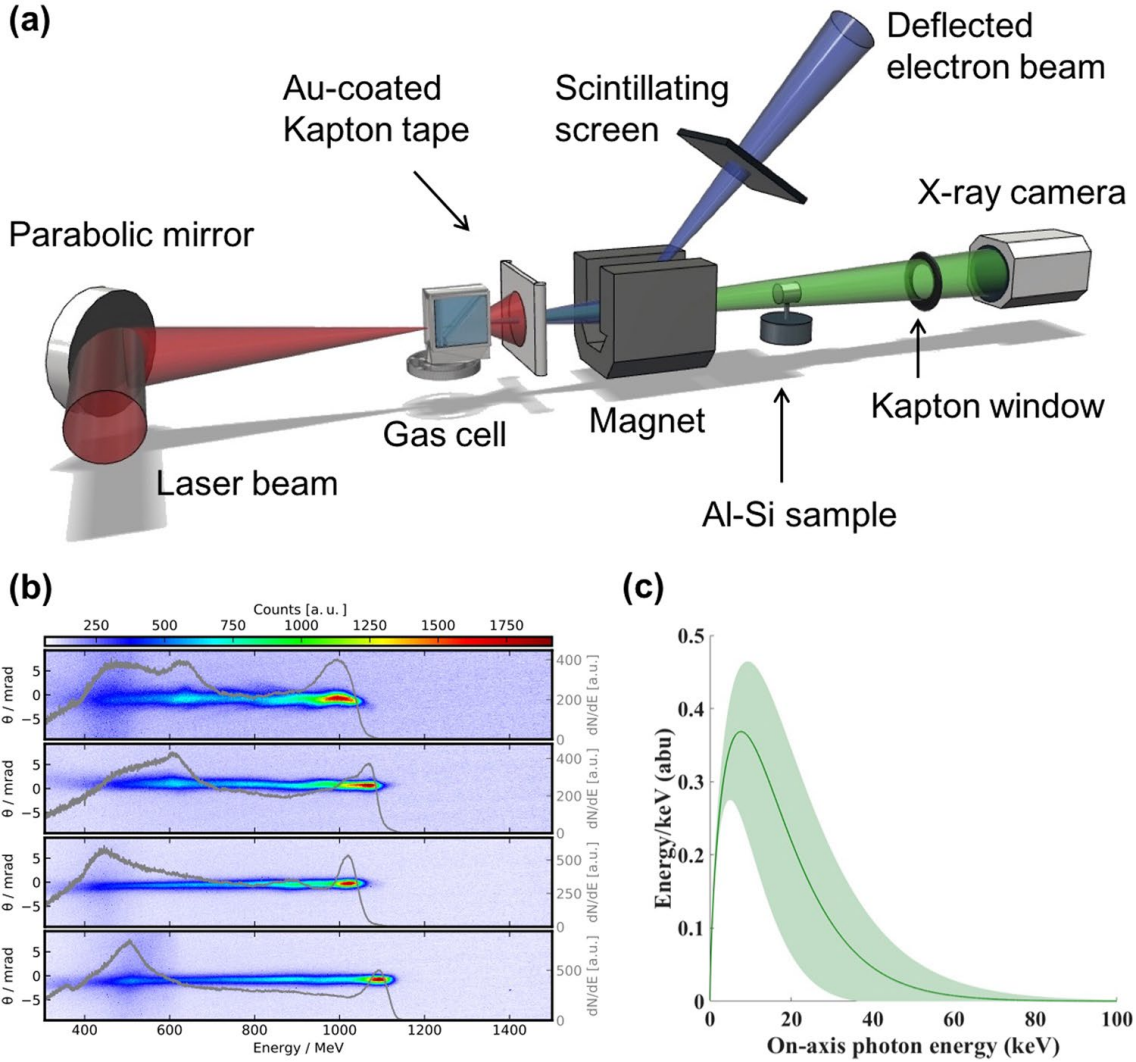
Experimental details for X-ray imaging using a laser wakefield accelerator. (**a**) Experimental layout. High energy electron and X-ray beams were produced by focusing the beam into a two-stage gas cell (see *Methods*). Gold-coated Kapton tape was used to block the laser pulse following the interaction, and was replaced on each shot. A 1 T magnet was used to disperse the electron beam onto a scintillating LANEX screen, from which the electron beam was imaged using a CCD camera. Betatron X-rays passed through the Al-Si sample, which was mounted on a rotation and translation stage at a distance of 19.3 cm from the source. Measurements were made through a kapton vacuum window onto an Andor iKon 2048 × 2048 pixel CCD camera at a distance of 410 cm from the Al-Si sample. (**b**) Samples of typical electron beams with a quasi-monoenergetic peak energy and broad low-energy tails. These measurements were obtained at the same experimental conditions as the phase contrast images and betatron spectrum. Electron beam divergence is plotted on the left axis and a line-out of the electron number density (right axis) is overlaid. (**c**) A best-fit to the betatron X-ray spectrum from an Andor iKon X-ray camera was obtained using a 9-element filter array (see *Methods*). Shaded error bars reflect the uncertainty in the critical energy over many shots due to shot-to-shot fluctuations in electron energy.

LWFA experiments were conducted using the Gemini laser at the Science and Technology Facilities Council (STFC), Rutherford Appleton Laboratory (RAL). The 40 fs FWHM laser pulse was focused using an *f*/40 parabolic mirror into a gas cell producing an electron beam. A schematic of the experimental setup at the Gemini laser system is given in Fig. 1(a). 3D printed two-stage gas cells have been shown to improve the stability, divergence and energy spread of LWFA electron beams^31^, therefore a two-stage gas cell with a 3 mm first stage for injection and a 2–21 mm variable length second stage was employed in this experiment (see *Methods*). Plasma density was controlled by altering the pressure of the gas supply of each individual stage, and density measurements were made using Stimulated Raman Side Scattering (see *Methods*). The plasma density corresponding to the optimum betatron spectrum was *n*_*p*_ = (4.1 ± 0.45) × 10^18^ cm^−3^ in both stages at a length of 15.5 mm. For these densities, electron beams with average peak energies of (1000 ± 150) MeV were produced. Example electron beams are shown in Fig. 1(b), with a superimposed line-out of the spectrum, indicating a quasi-monoenergetic peak and a broad low-energy tail. Further discussion of electron spectra and beam stability is presented in *Methods*. The X-ray beam, which was assumed to be synchrotron-like as shown in Fig. 1(c), contained 1.94 ± 1.24 × 10^8^ photons above 5 keV, and is estimated to have a source size smaller than 3 *μ*m, as discussed in *Results*. The LWFA X-ray beam has been found in similar experiments to have divergence on the order of a few millirads^15,17,18^ and femtosecond duration^16,19^. The electron and X-ray measurements shown in Fig. 1(b,c) were not obtained simultaneously, but were taken at identical experimental conditions. In these experiments, the Al-Si sample was 19.3 cm away from the betatron source and an X-ray CCD camera with pixel size of 13.5 *μ*m and a 100 ms exposure time was located 410 cm behind the sample (see *Methods*). A total of 136 single-shot images were acquired and no reconstructions were applied.

## Results

Al-Si samples for phase contrast imaging were prepared by the Materials Preparation Center at Ames Laboratory, with a composition of 50 wt% Si for the LWFA experiment and 30 wt% for the SLS experiment. Although the Al-Si sample used in the LWFA experiment had 20% more Si than that used in the SLS experiment, this excess Si is associated not with the Al-Si eutectic but rather the primary (*i*.*e*., pro-eutectic) Si phase. The larger mass fraction of this primary Si phase in the Al-Si alloy used in these experiments clouded the field-of-view in the X-ray images, limiting the eutectic — which is last to solidify — to a smaller region of the sample. However, this has little to no bearing on the development of the eutectic microstructure. Both alloys were cast in the exact same manner, and thus have comparable lamellar spacings (see *Methods*). For both experiments, the samples were machined into cylindrical samples of 1 mm thickness.

**Figure 2:**
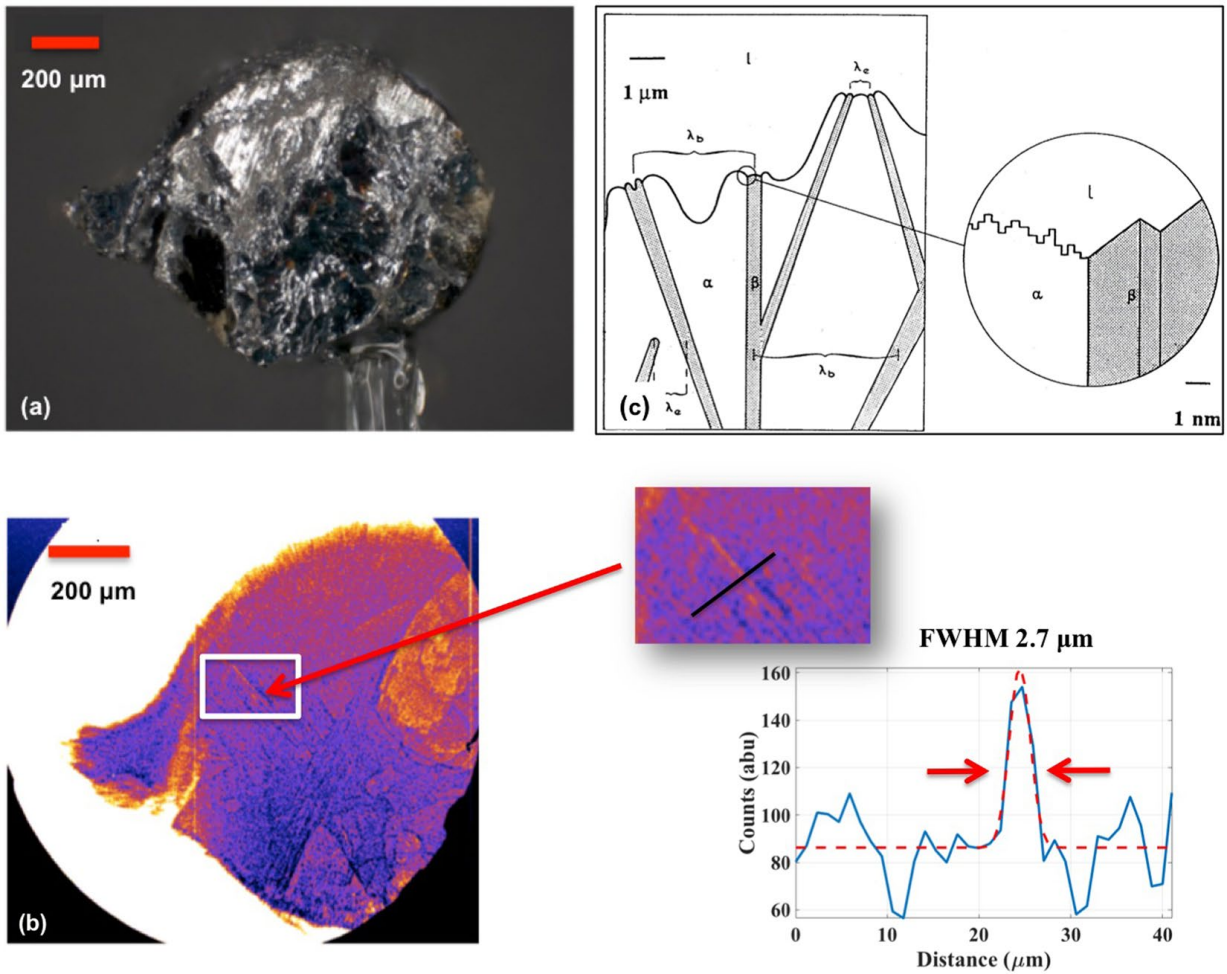
Al-Si sample investigated using a LWFA X-ray source. (**a**) Optical microscope image of the Al-Si cylindrical sample imaged in LWFA experiments. (**b**) X-ray phase contrast image obtained with a LWFA, revealing a lamellar microstructure with an interphase spacing on the order of 1–3 *μ*m. A line-out from a region of interest in the phase contrast image is shown, indicating 2.7 ± 0.3 *μ*m as an upper bound on the resolving power of this method. (**c**) A schematic showing growth of irregular eutectics where *β* represents the faceted phase (e.g., Si), *α* is the non-faceted, higher volume fraction phase (e.g., Al), and *l* is the melt ahead of the interface. The microstructure is deemed irregular due to the difficulty or “stiffness” in changing the growth direction of the faceted phase. The inset shows the atomically diffuse *α* phase and the defect growth mechanism for the faceted *β* phase. Retrieved with permission from ref.^73^.

A microscope image of the 1 mm diameter machined sample is shown in Fig. 2(a) alongside an example image of the Al-Si microstructure obtained using X-rays from a LWFA in Fig. 2(b). The LWFA projection image was obtained using a nearly 22× magnification, and the banded or lamellar structure can be observed in the zoomed-in image, from which a line-out indicates that the LWFA source is successfully resolving features smaller than 3 *μ*m. The resolution of these images is determined by the geometry of the imaging system, as discussed in *Methods*. The observed microstructure is consistent with that predicted for irregular eutectics, in which the lamellar spacing can be as fine as 1 *μ*m (Fig. 2(c)). In this idealized schematic, the faceted phase *β* (e.g., Si) and the non-faceted phase *α* (e.g., Al) are shown, growing in a non-periodic manner into the liquid.

The quality of the SLS and LWFA projection images were compared according to two metrics: image sharpness and resolution. Image sharpness is closely related to the fineness of the resolvable details in an image (X-ray projection microscopic images in this case). An algorithm developed by Shaked and Tastl^32^ was used to determine the overall sharpness of an image (see *Methods*). Spatial resolution was compared using a Fourier-based criterion^33^ on raw projection images obtained *via* a LWFA source and the TOMCAT beamline at the SLS. In this analysis, image quality was computed for the interior regions of phase contrast images to compare areas of highest resolution.

**Figure 3:**
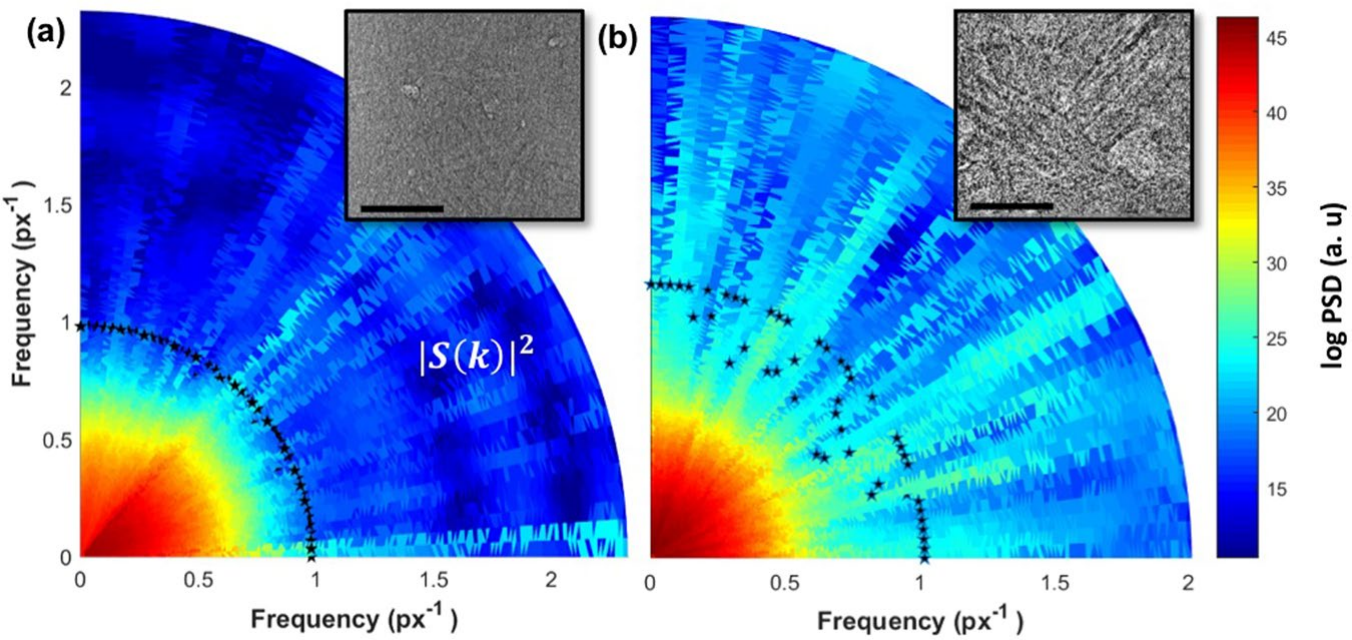
Measurement of the spatial resolution criterion for line profiles oriented from 0° to 90°. The spatial resolution criterion is projected onto polar plots in (**a**) SLS and (**b**) LWFA projection images. Projection images are shown as insets. |*S*(*k*)|^2^ is the spectral power of the detected signal. Raw images were resized to match the dissimilar pixel resolutions for SLS and LWFA images, and PSD analysis was performed on projection images with equalized intensity histograms. For both cases, spatial frequencies are given in units of inverse pixels. The LWFA projection image has a spatial resolution that is comparable to the spatial resolution in the SLS projection image, as evidenced by the close to equal k_*res*_ values of 1.017 ± 0.01 px^−1^ and 0.98 ± 0.01 px^−1^ in the LWFA and SLS images, respectively. Stars represent the *k*_*res*_ spatial frequency value obtained along an arbitrary line in the projection image. Scale bar measures 70 *μ*m.

Normalized sharpness estimates given in arbitrary units (1 ± 0.05 a.u. and 0.62 ± 0.05 a.u. for LWFA and SLS projection images, respectively) show that the LWFA projection images are comparable to the sharpness of projection images obtained at SLS (see *Methods*). In addition, Fig. 3 shows the calculation of the spatial resolution, where |*S*(*k*)|^2^ is the spectral power of the detected signal and *k*_*res*_ is the maximum spatial frequency when the spectral power is twice the noise level (see *Methods*). The power spectral density (PSD) conveys the strength of the intensity variation in the image pixels as a function of frequency; it indicates the frequencies at which intensity variations are strong and those at which the variations are weak. In other words, the high frequency wavenumber for the PSD in the LWFA image is related to sharper variations in intensity values of the pixels in the image domain. Such variations occur in pixels near to an object edge, e.g., between different lamellae in the Al-Si eutectic.

The PSD shown in Fig. 3(a,b) have been calculated for line profiles in the images taken using the TOMCAT beamline and *via* LWFA, respectively. The PSD profiles, projected onto polar plots, were computed for lines arbitrarily drawn within the projection image at angles ranging from 0° to 90° with the horizon to ensure that the PSD over all pixel directions in the projection images were statistically represented. It can be observed that the LWFA image has a spatial resolution *x*_*res*_ which is comparable to the spatial resolution in the SLS image. According to the Wiener-Khintchine theorem^34^, the autocorrelation function is the Fourier transform of the power spectral density. Accordingly, Fig. 3(b) shows a slightly higher autocorrelation at long wavelengths as evidenced by a higher *k*_*res*_ value of 1.017 ± 0.01 px^−1^ compared to 0.98 ± 0.01 px^−1^ for the SLS image, where LWFA images have been rescaled to the effective pixel value for proper comparison. Errors in the measurement of image resolution arise from the absence of normalization by white- and dark-field images for the LWFA projections (Fig. 3(b)), which ultimately lead to intensity inhomogeneities on the detector plane.

**Figure 4:**
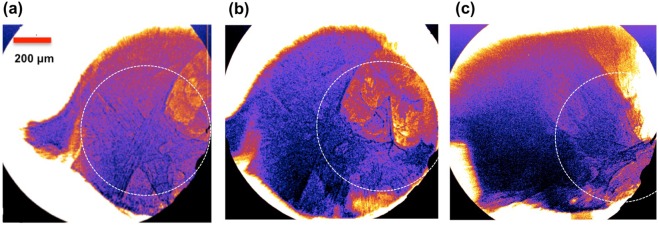
Blurring of LWFA X-ray images due to finite betatron emission length. Three LWFA phase contrast images of the Al-Si sample are shown. In (**a**,**b**) the sample is at the same orientation perpendicular to the laser axis. In image (**b**) the sample has been translated horizontally by approximately 30 *μ*m. In (**c**) the sample has been rotated by 90 degrees about the vertical axis. Regions of sharpest resolution are circled with a dotted line, with a radius of approximately 600 *μ*m at highest focus. In all images, blurring can be observed on the order of a millimeter away from the central point due to the emission length of the betatron source. Highest resolution imaging is obtained along the axis of the electron beam; only this section of the image is used for resolution analysis. Blurring due to the emission length of the X-ray source is not unique to betatron sources, also occurring with conventional synchrotron beams, but is exacerbated by high magnification in cases where the full beam is used for imaging.

Beyond sharpness and resolution, another consideration in the practical application of LWFA for X-ray imaging is blurring due to the non-zero emission length of the betatron source^35^. Betatron emission is highest at the location of high energy electrons, yielding a very small source size on the order of a few *μ*m^11^. However, the emission length of a betatron source has been found to extend a few millimeters along the axis of laser propagation, resulting in blurring in X-ray images and decreased resolution^35,36^. This blurring can be observed in Fig. 4, where the image resolution is highest near the central axis of the X-ray beam (circled) and begins to blur towards the edges of the sample. It has also been found that the betatron emission length tends to increase with increasing plasma length^36^, therefore longer plasma lengths are associated with lower resolution away from the central axis of the laser beam. Additionally, instability in beam pointing can result in variation of the location of highest resolution. For a plasma cell of length 15.5 mm, as employed in these experiments, the emission length of the betatron source was found to be on the order of 5 mm. Image blurring is also a challenge with conventional synchrotron sources, where the emission length can be much longer (~m), versus ~mm for a LWFA source. However, the large divergence of the LWFA source makes this a concern when the full beam size is used for imaging. It is also important to note that blurring due to the emission length is exacerbated by high magnification. The relationship between plasma length and emission can inform optimization of the LWFA X-ray source for high resolution imaging.

**Figure 5:**
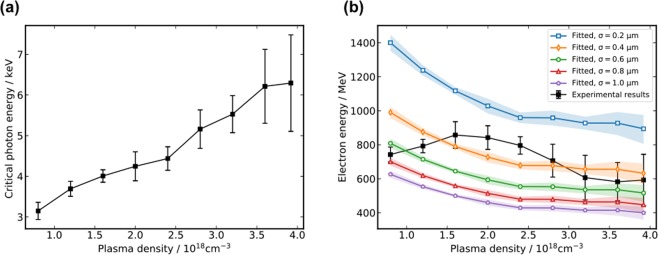
Critical energy of the LWFA betatron source. (**a**) Experimentally measured critical energy of the LWFA X-ray beam as a function of plasma density. (**b**) Theoretical predictions of the maximum electron energy corresponding to experimentally measured critical energy, shown for betatron source sizes of (0.2–1.0) *μ*m along with experimentally measured maximum electron energies in the resultant LWFA beam (black).

For high contrast imaging of features in dense materials the critical energy of the X-ray beam must be on the order of several keV. In this experiment, the critical energy of the resultant X-ray beam is determined by comparing the transmission through an array of different elemental filters (see *Methods*). The critical energy as a function of plasma density was found to increase with increasing plasma density, as shown in Fig. 5(a), reaching a maximum critical energy of nearly 10 keV. These results indicate that LWFA X-ray sources can provide a tunable X-ray source for phase contrast imaging.

The critical photon energy of a LWFA source is related to the maximum energy of the electron beam, *γ*, and the plasma density, *n*_*p*_, by^37–39^:

1 $${E}_{c}=\frac{3}{2}\hslash {\omega }_{\beta }K{\gamma }^{2}=\frac{3}{4}\hslash \frac{{e}^{2}}{{\varepsilon }_{0}{m}_{e}c}\frac{\sigma }{2}{n}_{p}{\gamma }^{2}$$

where *σ* = 2*r*_*β*_ is the approximate betatron source size and *r*_*β*_ is the amplitude of betatron oscillations. From equation (1) the electron energy can be retrieved from the measured critical photon energy, the plasma density *n*_*p*_ and an assumed source size *σ* using $\gamma \propto \sqrt{2{E}_{c}/{n}_{p}\sigma }$. Figure 5(b) shows the retrieved electron energies with measured plasma densities and fitted source size *σ* of (0.2–1.0 *μ*m). A plot of the experimentally measured peak electron energy is superimposed on retrieved electron energies, showing best agreement between theory and experimental data for betatron source size on the order of (0.4–1.0) *μ*m.

For comparison with experimental results it is important to note that the critical photon energy in equation (1) is mainly determined by the maximum electron energy achieved during acceleration because of the *γ*^2^ scaling. Therefore, the retrieved electron energies represent the maximum electron energies during the acceleration, which are not necessarily the same as those measured from the experiment. This is because for high plasma density (here, *n*_*p*_ > 1.2 × 10^18^ cm^−3^) the dephasing length is shorter than the gas cell length and electron beams will experience dephasing. Currently, information about electron dephasing cannot be captured experimentally in a single shot, however novel techniques employing a transverse density gradient may provide single-shot diagnostic information of the temporal evolution of the betatron X-ray spectrum and electron acceleration^40^.

## Discussion

Thus far, Al-Si eutectics have only been investigated *via* conventional synchrotron-based phase contrast tomography (PCT)^41–43^. PCT enables the study of weakly absorbing samples, as well as materials systems consisting of elements with similar atomic numbers. This is because variations in the real valued refractive index are several orders of magnitude larger than the imaginary component^44,45^. In order to recover the microstructure from projection images obtained *via* PCT, phase-retrieval algorithms are first applied to the projection images^46,47^. Subsequently, a projection algorithm (*e*.*g*., *filtered back projection*^48^) is used to reconstruct a three-dimensional (3D) map of the refractive index decrement (i.e., the difference between the sample’s index of refraction and that of air). Image segmentation of the PCT reconstructions is crucial for quantitative analysis of interfacial properties, *e*.*g*., orientations, velocities, curvatures, and *n*-point statistics^49,50^. However, sharp images taken at high resolution with sufficient contrast, such as those obtained with a LWFA source, can mitigate the challenges associated with low pass characteristics in projection images and in turn ease the data analysis process down-stream^41,51,52^,

From the projection images obtained in the LWFA experiment the microstructural details can be measured straightaway and throughout the sample volume owing to the fact that the projection images were reasonably sharp. In particular, the spacing between neighboring Si lamellae was measured to be between 10 and 90 *μm*. Using the Jackson-Hunt relationships modified for irregular eutectics^22,24,53,54^, the average lamellar spacing was correlated with an average growth rate and undercooling, estimated to be 0.35 ± 0.3 *μ*m/s and 0.13 ± 0.03 K, respectively (see *Methods*). The combination of the two solidification parameters gave rise to the eutectic microstructure observed in the LWFA projection images. Additionally, the lamellar morphology as shown in Fig. 3(b), inset, exhibits a flake-like Si phase that is commonly found in irregular eutectics of undoped or unmodified alloys. This morphology is to be fully expected given the high purity of the constituent Al and Si powders, as discussed in *Methods*. The eutectic Si flakes extend laterally by a process known as twin plane re-entrant edge mechanism (TPRE), which was first introduced by Wagner^55^ and Hamilton and Seindensticker^56^. Branching events between the Si flakes that are likewise facilitated by twins were also observed^57^. Altogether, these preliminary observations suggest the importance of growth twinning for the continued propagation of the faceted Si phase during solidification. A more conclusive argument for the growth mechanism of undoped and doped alloy samples cannot be made until a 4D (*i*.*e*., space- and time-resolved) assessment of the microstructure is performed, which is the focus of future research.

The resolution limits of phase-contrast X-ray imaging experiments are set by the source size and the imaging geometry, which determines the magnification of the system, and the detector pixel size. For synchrotron beamlines, such as TOMCAT, the source size is much bigger than the desired resolution, but the distance from the source to the sample is typically much larger than the distance from the sample to the detector, effectively resulting in a large demagnification factor of the source size. Therefore, the effective pixel size of the detector (which includes the optical magnification provided by the visible light microscope coupling the scintillator to the detector’s imaging chip) is the limiting factor for high-resolution imaging, and needs to be minimized for the highest possible resolution. Conversely, for LWFA sources, where the source size is much smaller than the pixel size of the detector, the high magnification in the X-ray imaging geometry reduces the resolution requirements of the detector. For high resolution phase contrast imaging, the conditions for detection of bright and dark phase contrast fringes are set by the detector resolution, and the bandwidth and size of the source^45^.

A comparison of experimental parameters used in the SLS and LWFA experiments presented here indicates that these sources have comparable geometric resolution limits and both satisfy the criteria for fringe detection (see *Methods*). However, our analysis of the projection images shown in Fig. 3 indicates that the LWFA source has slightly greater sharpness and spatial resolution for these conditions and is able to resolve micrometer-scale lamellar features. The reason for resolution loss in the SLS projection image is likely due to vibrations in the experimental setup. At the time of the experiments, the relative sample to detector position could vibrate at an amplitude of up to 0.5–1 *μ*m consequently resulting in a blurring of the projection images over the 500 milliseconds exposure time. Conversely, although LWFA experiments are prone to similar instabilities, the femtosecond timescale of the betatron source enables ultrafast imaging. Therefore, single-shot LWFA images are not subject to motion blur. In this way, the visibility of small-scale features such as lamellae is enhanced. It is also worth noting that the conditions for detecting phase contrast fringes for the LWFA experiments set an upper bound of 1 *μ*m on the source size, indicating that betatron sources may be much smaller than previously noted.

Our results indicate that betatron X-rays from LWFA can be competitive with conventional synchrotron sources for the characterization of eutectic alloys and solid density materials. This opens the door to high-resolution materials diagnostics using laser-based sources, without needing to visit a synchrotron facility. Indeed, projection images of the Al-Si sample obtained using LWFA betatron X-rays were of comparable sharpness and spatial resolution to projection images obtained at SLS. Fine details of the lamellar microstructure were clearly resolved in LWFA projection images (Fig. 3(b) inset), indicating an upper bound of 2.7 *μ*m on the resolving power of this method. Furthermore, the phase contrast spatial resolution criteria indicate that the LWFA source size may be much smaller than a micrometer, which is corroborated by the theoretical scaling of the betatron energy with plasma density in Fig. 5(b) in which the retrieved electron energy was most closely fit assuming betatron source sizes on the order of (0.4–1.0) *μ*m. However, it is important to note that the enhanced spatial resolution reported in this paper is specific to the experimental conditions of these experiments, and that neither of the two experiments was optimized to obtain the ultimate spatial resolution. The ultrashort exposure time of betatron sources may also provide improved spatial resolution by enabling imaging on a timescale shorter than the frequency of vibrations in experimental setups.

As mentioned in the *Introduction*, one area in which we can demonstrate significant near-term impact of these LWFA sources is through the use of betatron X-rays as a diagnostic tool for real-time monitoring of additive manufacturing. In recent years, additive manufacturing has seen tremendous growth due to developments in processes and materials, as well as a greater understanding of the underlying design principles. It already has huge societal impacts through the ability to produce cheaper and customizable products, such as artificial hips and lightweight aircraft components^58–60^. As-solidified parts have traditionally been characterized by examining their microstructures following manufacturing, however such *post mortem* approaches lack the capability of tracking the interfacial dynamics during the solidification process. In fact, it is well known that quenching distorts the morphology of the solid-liquid interfaces, and thus the micrographs collected following manufacturing do not depict those same interfaces that are present during laser-aided processing. Moreover, the US National Institute of Science and Technology’s “Measurement Science Roadmap for Metal-Based Additive Manufacturing” identifies *in situ* process monitoring and metrology *as a key barrier to additive manufacturing implementation*^61^. To address this confounding issue, a few investigators have recently employed synchrotron-based X-ray microtomography to track the microstructural evolution as a function of time^27,62^. High-speed synchrotron hard X-ray imaging on the nanosecond timescale has recently been demonstrated^20^, however LWFA sources offer temporal resolution on the order of femtoseconds^16,63,64^. The realization of high-repetition rate laser drivers for LWFA^65–67^ could enable dynamic measurements on an ultra-short timescale. Therefore, the micrometer-scale spatial resolution demonstrated in this paper, combined with femtosecond temporal resolution and high repetition capabilities, indicate that LWFA sources could be used for high-resolution dynamics measurements on an ultra-short timescale.

## Methods

### Laser

The LWFA experiments were carried out on the Gemini laser facility at the Science and Technology Facilities Council (STFC), Rutherford Appleton Lab (RAL), UK. The pulse for laser wakefield acceleration had a FWHM pulse duration of 40 ± 3 fs, a central wavelength of 800 nm, and was linearly polarized. The laser pulse with energy of 16.4 ± 0.6 J before the compressor, yielding approximately 8.4 ± 0.6 J on target. The pulse was focused by an *f*/40 off-axis parabolic mirror with a focal length of 6 m to a 1/*e*^2^ focal spot of 36.3 ± 0.8 *μ*m, yielding a peak intensity of 1.0 × 10^19^ W/cm^2^ (*a*_0_ = 2.0) within the FWHM of the focal spot.

### Gas target

**Figure 6:**
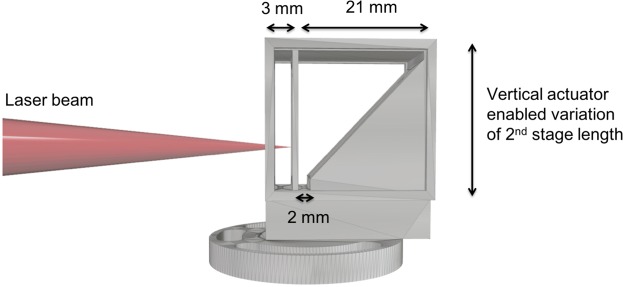
CAD model of the variable length two-stage gas cell used in LWFA experiments. A two-stage gas cell with a 3 mm first stage for ionization injection and a variable length second stage was used. A 45° wall in the second stage enabled variation of the length of the second stage (between 2 to 21 mm) using linear motor controls to vary the vertical position of the cell.

The laser was focused into a 3D printed two-stage length gas cell, with mixed gas (2% nitrogen and 98% helium) in the first stage and helium gas in the second stage. The length of the first stage in the gas cell, used for ionization injection, was 3 mm, and the length of the second stage was varied between 2 and 21 mm using linear actuators to change the position of the laser relative to a 45° exit wall, as shown in Fig. 6. The thickness of the entrance and stage divider walls were 1 mm, and the exit wall was 2 mm thick. The plasma density was controlled by altering the backing pressure of the gas supply. Plasma density measurements were made using calibrated Stimulated Raman Side Scattering measurements^68^ and yielded *n*_*p*_ = (4.1 ± 0.45) × 10^18^ cm^−3^ in both stages at a cell length of 15.5 mm.

### Electron and X-ray beam characterization

**Figure 7:**
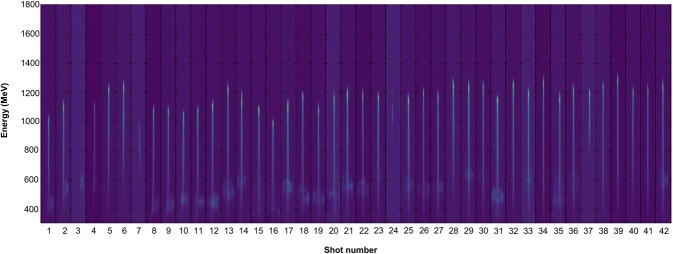
Electron beam profiles. Electron spectra obtained for 42 consecutive laser shots at identical experimental conditions.

A 1 T magnet was used to disperse the electron beam onto a scintillating LANEX screen, from which the electron beam was imaged using a CCD camera. Particle tracing was performed with the measured magnet field map to calculate the electron energy as a function of the position on the lanex screen. A series of electron spectra from consecutive shots at identical experimental conditions is presented in Fig. 7, indicating good shot-to-shot reproducibility of accelerated beams at a plasma density of *n*_*p*_ = (4.1 ± 0.45) × 10^18^ cm^−3^. The average peak energy of the beams shown in Fig. 7 was (1200 ± 50) MeV, but for all spectra at these conditions the average peak energy was (1000 ± 150) MeV. Low energy features on the beams are likely untrapped energetic electrons, which have been found to form ring structures^69,70^.

The X-ray beam was collected by an on-axis X-ray camera (model: Andor iKon-L SY DW936 BR-DD) with a 250 *μ*m beryllium filter, placed 429.3 cm away from the source. In front of the X-ray camera a 9-element filter array composed of various materials with different K-edges was placed to characterize the X-ray spectral distribution^11,15^. The thickness of each filter element can be found in Table 1. The signal counts on camera can be estimated as^71^:

2 $${N}_{i}=\eta \,{\int }_{{E}_{min}}^{{E}_{max}}\,S(E,{E}_{crit})Q(E){T}_{i}(E)dE$$

where *η* is a constant coefficient, $S(E,{E}_{crit})\sim {(E/2{E}_{crit})}^{2}{K}_{2/3}^{2}(E/2{E}_{crit})$ is the on-axis synchrotron spectrum with a critical energy of *E*_*crit*_, *Q*(*E*) is the quantum efficiency of the camera, *T*_*i*_ is the overall transmission of the filter *i* with the consideration of attenuation of other materials in the beam path. Fitting equation (2) with the measured signal counts on the camera for all the filters gives a best fitted *E*_*crit*_.

**Table 1:** Thickness of filter array elements.

Material	Nb	Mo	Cu	Zn	Fe	Co	Sc	Ti	Pb
Thickness (*μ*m)	24.5	20.0	9.2	10.0	5.6	5.4	26.7	17.3	503.8

It is important to note that characterizations of the electron and X-ray beams were not obtained from a single experimental day, but were compiled using data from experimental runs at the same conditions as the measurements that yielded the phase contrast images of complex microstructures presented in this paper. Simultaneous measurements of the electron beam with phase contrast imaging was not possible in these experiments due to the necessity of an additional “kicker” magnet to protect the sample by further deflecting the electron beam.

### Image sharpness and resolution

The SLS projection image was normalized according to the standard procedure for synchotron experiments using dark images and flat-field corrections as follows:

3 $${\rm{Normalized}}\,{\rm{SLS}}\,{\rm{image}}=\tfrac{(({\rm{Raw}}\,{\rm{SLS}}\,{\rm{projection}}\,{\rm{image}})-({\rm{average}}\,{\rm{of}}\,{\rm{21}}\,{\rm{dark}}\,{\rm{shots}}))}{(({\rm{average}}\,{\rm{of}}\,{\rm{51}}\,{\rm{flat}}\,{\rm{shots}})-({\rm{average}}\,{\rm{of}}\,{\rm{21}}\,{\rm{dark}}\,{\rm{shots}}))}$$

No such normalizations were applied to LWFA images.

An algorithm developed by Shaked and Tastl^32^ was used to determine the overall sharpness of an image. Here, their global single parameter sharpness model is used, implemented as the ratio between the output energy of an ideal high pass filter and an ideal band pass filter^32^, and described by

4 $${\rm{Sharpness}}=\frac{{\int }_{\bar{\xi }\varepsilon H}\,|M(\bar{\xi }){|}^{2}d\bar{\xi }}{{\int }_{\bar{\xi }\varepsilon B}\,|M(\bar{\xi }){|}^{2}d\bar{\xi }}$$

where the image is indicated by *m*(*x*, *y*) and the Fourier transform of the image by *F*(*m*) = *M*(*ξ*_*x*_, *ξ*_*y*_), the Cartesian frequency coordinates are defined as $\bar{\xi }=({\xi }_{x},{\xi }_{y})$, and *H* and *B* are the high and low-band pass frequency ranges, respectively. The images were initially resized to match the dissimilar pixel resolutions (0.74 *μ*m and 0.61 *μ*m for SLS and LWFA experiments, respectively), and the intensity histogram in each image was scaled to lie within the same intensity range. Subsequently, a 2D high pass filter and 2D band pass filter were applied to the 2D Fourier transform of each image matrix and the image sharpness was calculated according to equation (4).

A Fourier-based spatial resolution criterion^33^ was used on projection images obtained *via* a laser-wakefield accelerator system and the TOMCAT beamline at the Swiss Light Source. The power spectral density (PSD) profiles of lines arbitrarily drawn within the projection image at angles ranging from 0° to 90° with the horizon are computed. This was done to ensure that the power spectral density over all pixel directions in the projection images were statistically represented. The PSD values can then be projected onto polar plots to reveal the power spectral distribution at varying angular positions within the image. The PSD converges to a value defined as the “noise baseline” obtained in our calculations by taking the mean of the last fifty (50) power spectral density elements in the array of PSDs. According to the criterion put forward by ref.^33^, spatial resolution is computed by taking twice the value of the PSD at the noise baseline, and matching this value to the corresponding maximum spatial frequency, *k*_*res*_^33^. The spatial resolution *x*_*res*_ is related to the wavenumber *k*_*res*_ by:

5 $${x}_{res}=\frac{2\pi }{{k}_{res}}$$

### Resolution limits

**Figure 8:**
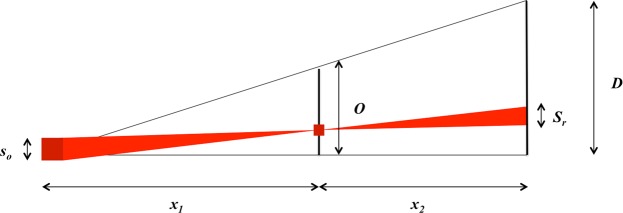
Geometric layout of a X-ray illumination setup without optics. The distance from the source, *s*_*o*_ to the object, *O*, is *x*_1_, and the distance from the object to the detector, *D*, is *x*_2_.

The resolution in a lens-less X-ray image setup is determined by the imaging geometry and the detector, as shown in Fig. 8. For a source of size *s*_*o*_ at a distance of *x*_1_ from an object, *O*, an image is formed at the detector, *D*. The distance from the object to the detector is *x*_2_. In this configuration there are two limitations on the resolution dictated by source size and the detector resolution, both of which depend on the magnification of the system.

The geometric magnification of the system is related to the distances between the source and the object and the object and the detector using similar right angle triangles: *M* = *D*/*O* = (*x*_1_ + *x*_2_)/*x*_1_. The transverse projection of a point in the object onto the detector determines the source-size limited resolution, *S*_*r*_ = *s*_*o*_*x*_2_/*x*_1_. At the object plane, the resolution limit of the source is given by *S*_*r*_/*M* = *s*_*o*_*x*_2_/(*x*_1_ + *x*_2_). The resolution limit of the detector is set by the pixel size, *p*, and therefore the lower bound on detector resolution is *D*_*r*_ = *p*/*M* = *px*_1_/(*x*_1_ + *x*_2_). The total resolution, *r*, can be considered as the 2-norm of these limits^72^:

6 $$r={({(\frac{{s}_{o}{x}_{2}}{{x}_{1}+{x}_{2}})}^{2}+{(\frac{p{x}_{1}}{{x}_{1}+{x}_{2}})}^{2})}^{1/2}$$

The conditions for detecting fringes due to phase contrast imaging are set by the detector resolution, the wavelength bandwidth and the source size^45^. The X-ray detector must have sufficient resolution to resolve separate fringes, where the fringe spacing is given by $z\simeq \sqrt{{x}_{2}\lambda }$ where *λ* is the wavelength of the critical energy of the source, given in Table 2. Using the values in Table 2, it is clear that this condition is satisfied in both the SLS and LWFA experiments. The condition on the longitudinal coherence of the source is given by $\Delta \lambda /\lambda \ll 2$. This conditions is rather weak and therefore can be assumed to be automatically satisfied for both sources^45^.

**Table 2:** Comparison of resolution limits in X-ray imaging between the Swiss Light Source (SLS) and LWFA X-ray sources generated using the Gemini Laser at the Rutherford Appleton Lab (RAL).

	SLS	LWFA
Source size, FWHM (*s*_0_)	127 *μ*m (H) × 38 *μ*m (V)	<2.7 *μ*m (1 *μ*m assumed)
Detector pixel size (*p*)	0.75 *μ*m	13.5 *μ*m
Source to sample (*x*_1_)	2000 cm	19.3 cm
Sample to detector (*x*_2_)	11 cm	410 cm
Magnification (*M*)	$\simeq$1.01	22.2
Source size limited resolution at the object plane (*S*_*r*_/*M*)	0.69 *μ*m × 0.21 *μ*m	0.96 *μ*m
Detector resolution limit (*p*/*M*)	0.74 *μ*m	0.61 *μ*m
Total geometric resolution (*r*)	1.0 *μ*m × 0.76 *μ*m	1.1 *μ*m
Phase contrast detector limit (*z*)	2.2 *μ*m	21 *μ*m
Critical energy (*E*_*c*_)	28 keV	11.2 keV
Wavelength (*λ*)	4.4 × 10^−11^ m	1.1 × 10^−10^ m
Phase contrast source size limit (*y*)	$\ll$400 *μ*m	$\ll$1 *μ*m

Errors on all measurements are approximately 10%.

The final condition on the resolution of phase-contrast imaging is set by the lateral coherence of the source, or the source size. A finite source size can be considered as a pair of point sources, separated by a finite distance, *y*. These two sources will each produce fringes at the detector. The shift between these fringes can result in blurring and decreased resolution. The limit on the source size for resolving individual fringes is given by $y\ll {x}_{1}\sqrt{\lambda /{x}_{2}}$. The parameters above are tabulated for the SLS and the LWFA generated X-ray source in Table 2.

### Materials

The Al-Si targets for phase contrast imaging were prepared by the Materials Preparation Center at Ames Laboratory (Ames, IA, USA). High-purity powders (99.99% Al and 99.9999% Si) were prepared by melting three times in a low-pressure argon (Ar) atmosphere to mix and degas the melt. In this way, castings in the shape of buttons were produced with a composition of 50 wt% Si for the Rutherford Appleton Laboratory (RAL) experiment and 30 wt% for the Swiss Light Source (SLS) experiment. The as-cast buttons were machined into cylindrical samples of 1 mm diameter using Electrical Discharge Machining (EDM).

### Lamellar spacing, growth rate, and undercooling

In measuring the growth rate and undercooling based on the interflake lamellar spacing, the following relationships based on the modified Jackson – Hunt eutectic theory^24,53,54^ were employed:

7 $${\lambda }^{2}V={K}_{1}\,{\rm{and}}\,\Delta T={K}_{2}{V}^{0.53}$$

where *K*_1_ = 780.04 and *K*_2_ = 0.24. To solve for the growth rate, *V*, and undercooling, Δ*T*, the lamellar spacings *λ* were measured from the LWFA projection images as input. Values for *λ* ranged from approximately 10 ± 0.5 *μ*m to 90 ± 0.5 *μ*m. Errors in the measurement of the lamellar spacings arise due to the fact that only *projected* spacings can be measured in the projection images and may not represent the *true* spacing between lamellae, depending on whether the lamellar are tilted with respect to the plane perpendicular to the X-ray beam. Consequently, the growth rate was found to vary between 0.1 ± 0.3 *μ*m/s to 1.2 ± 0.3 *μ*m/s while the undercooling was found to vary between 0.065 ± 0.03 K to 0.25 ± 0.03 K. It is anticipated that a 3D microstructural analysis *via* phase contrast X-ray tomography in the laser wakefield accelerator setup could aid in the refinement of calculations of the lamellae spacing, growth rate, and undercooling and further enhance our understanding of the detailed morphology and topology of the Al-Si eutectic microstructure and other related alloys.

## Data Availability

The authors confirm that all of the data used in this study are available without restriction. Data can be obtained by contacting aehuss@umich.edu.
