# Author Correction: Laser-wakefield accelerators for high-resolution X-ray imaging of complex microstructures

**DOI:** 10.1038/s41598-019-55304-6

**Published:** 2020-01-10

**Authors:** A. E. Hussein, N. Senabulya, Y. Ma, M. J. V. Streeter, B. Kettle, S. J. D. Dann, F. Albert, N. Bourgeois, S. Cipiccia, J. M. Cole, O. Finlay, E. Gerstmayr, I. Gallardo González, A. Higginbotham, D. A. Jaroszynski, K. Falk, K. Krushelnick, N. Lemos, N. C. Lopes, C. Lumsdon, O. Lundh, S. P. D. Mangles, Z. Najmudin, P. P. Rajeev, C. M. Schlepütz, M. Shahzad, M. Smid, R. Spesyvtsev, D. R. Symes, G. Vieux, L. Willingale, J. C. Wood, A. J. Shahani, A. G. R. Thomas

**Affiliations:** 1 Center for Ultrafast Optical Science, University of Michigan, Ann Arbor, MI 48109-2099 USA; 2 Department of Materials Science and Engineering, University of Michigan, Ann Arbor, MI 48109-2099 USA; 3 Physics Department, Lancaster University, Lancaster, LA1 4YB UK; 4 The Cockcroft Institute, Keckwick Lane, Daresbury, WA4 4AD UK; 5 The John Adams Institute for Accelerator Science, Imperial College London, London, SW7 2AZ UK; 6 Lawrence Livermore National Laboratory, NIF and Photon Sciences, Livermore, CA 94550 USA; 7 Central Laser Facility, STFC Rutherford Appleton Laboratory, Didcot, OX11 0QX UK; 8 Diamond Light Source, Harwell Science and Innovation Campus, Fermi Avenue, Didcot, OX11 0DE UK; 9 Department of Physics, Lund University, P.O. Box 118, S-22100 Lund, Sweden; 10 York Plasma Institute, Department of Physics, University of York, York, YO10 5DD UK; 11 SUPA, Department of Physics, University of Strathclyde, Glasgow, G4 0NG UK; 12 Helmholtz-Zentrum Dresden-Rossendorf, Bautzner Landstraße 400, 01328 Dresden, Germany; 13 Technische Universität Dresden, 01062 Dresden, Germany; 14 Institute of Physics of the ASCR, 182 21 Prague, Czech Republic; 15 GoLP/instituto de Plasmas e fusão nuclear, Instituto Superior Técnico, U.L., Lisboa, 1049-001 Portugal; 16 Swiss Light Source, Paul Scherrer Institute, CH-5232 Villigen, Switzerland; 17 ELI Beamlines, Institute of Physics of the ASCR, 182 21 Prague, Czech Republic

Correction to: *Scientific Reports* 10.1038/s41598-019-39845-4, published online 01 March 2019

In the original version of this Article, K. Falk and M. Smid were incorrectly affiliated with ‘ELI Beamline, Institute of Physics of the ASCR, Na Slovance 2, Prague, 182 21, Czech Republic’. The correct affiliations are listed below.

K. Falk:

Helmholtz-Zentrum Dresden-Rossendorf, Bautzner Landstraße 400, 01328 Dresden, Germany.

Technische Universität Dresden, 01062, Dresden, Germany.

Institute of Physics of the ASCR, 182 21 Prague, Czech Republic.

M. Smid:

Helmholtz-Zentrum Dresden-Rossendorf, Bautzner Landstraße 400, 01328 Dresden, Germany.

ELI Beamlines, Institute of Physics of the ASCR, 182 21 Prague, Czech Republic.

These errors have now been corrected in the PDF and HTML versions of the Article.

